# High mortality rates in men initiated on anti-retroviral treatment in KwaZulu-Natal, South Africa

**DOI:** 10.1371/journal.pone.0184124

**Published:** 2017-09-13

**Authors:** Kogieleum Naidoo, Razia Hassan-Moosa, Nonhlanhla Yende-Zuma, Dhineshree Govender, Nesri Padayatchi, Halima Dawood, Rochelle Nicola Adams, Aveshen Govender, Tilagavathy Chinappa, Salim Abdool-Karim, Quarraisha Abdool-Karim

**Affiliations:** 1 Centre for the AIDS Programme of Research in South Africa (CAPRISA), Durban, KwaZulu-Natal, South Africa; 2 MRC-CAPRISA HIV-TB Pathogenesis and Treatment Research Unit, Doris Duke Medical Research Institute, University of KwaZulu-Natal, Durban, KwaZulu-Natal, South Africa; 3 Mailman School of Public Health, Department of Epidemiology, Columbia University, New York, New York, United States of America; University of Pittsburgh Centre for Vaccine Research, UNITED STATES

## Abstract

In attaining UNAIDS targets of 90-90-90 to achieve epidemic control, understanding who the current utilizers of HIV treatment services are will inform efforts aimed at reaching those not being reached. A retrospective chart review of CAPRISA AIDS Treatment Program (CAT) patients between 2004 and 2013 was undertaken. Of the 4043 HIV-infected patients initiated on ART, 2586 (64.0%) were women. At ART initiation, men, compared to women, had significantly lower median CD4+ cell counts (113 vs 131 cells/mm^3^, p <0.001), lower median body mass index (BMI) (21.0 vs 24.2 kg/m^2^, p<0.001), higher mean log viral load (5.0 vs 4.9 copies/ml, p<0.001) and were significantly older (median age: 35 vs. 32 years, p<0.001). Men had higher mortality rates compared to women, 6.7 per 100 person-years (p-y), (95% CI: 5.8–7.8) vs. 4.4 per 100 p-y, (95% CI: 3.8–5.0); mortality rate ratio: 1.54, (95% CI: 1.27–1.87), p <0.001. Age-standardised mortality rate was 7.9 per 100 p-y (95% CI: 4.1–11.7) for men and 5.7 per 100 p-y (95% CI: 2.7 to 8.6) for women (standardised mortality ratio: 1.38 (1.15 to 1.70)). Mean CD4+ cell count increases post-ART initiation were lower in men at all follow-up time points. Men presented later in the course of their HIV disease for ART initiation with more advanced disease and experienced a higher mortality rate compared to women.

## Introduction

In South Africa, an estimated 6.2 million people are currently living with HIV infection [1]. South Africa has been successful in implementing the largest anti-retroviral programme in the world with more than 3 million HIV infected people currently on treatment [2]. Despite this, high HIV incidence rates continue to occur, and HIV related morbidity and mortality dominates as the main cause of hospitalization and premature death for the last 2 decades [3].

In order to achieve the UNAIDS 90-90-90 goals for epidemic control and enhance individual and population level impact of public access anti-retroviral therapy (ART), detailed understanding of gaps in access to treatment services and factors that contribute to poor HIV treatment outcomes including ongoing mortality, is imperative. Data from other countries are inconsistent and contradictory, with some studies demonstrating gender imbalances in access to HIV treatment services and in clinical outcomes among patients on ART [4,5], and others demonstrating poorer clinical outcomes in women [6–8] or no gender difference in immunological response or mortality [9].

Notwithstanding the higher burden in women and 5–7 year earlier acquisition of HIV infection in adolescent girls and young women between the ages of 15–24 years [10], men are known to be poor utilizers of health care services [11] and characteristically present at a late stage of disease [12]. Women, are the predominant users of public sector ART [13], with reports from South Africa indicating that up to 60% of eligible women received ART compared to 41% of eligible men by mid-2011 [14]. This reflects the reality that infected women are linked to care through routinely offered HIV testing when utilizing antenatal and other sexual reproductive health services; an option that is not available for men.

A South African study assessing the sustainability of task shifting in a rural primary care clinic demonstrated that 68.8% of those accessing ART services between 2004 and 2010 (p < 0.05) were women [15], and those men that access ART are less likely to be retained in care [16].

In a country with the largest ART rollout program, it is important to understand who the current utilizers of HIV treatment services are, as this will help inform efforts aimed at reaching those currently not being reached. This study sought to investigate whether there were gender and age differences in those accessing HIV care services and clinical outcomes in those initiated on ART among urban and rural patients utilizing a free HIV treatment program in KwaZulu-Natal, South Africa.

## Methods

### Study design

We undertook a retrospective chart review of ambulant HIV infected, ART naïve adult patients enrolled into the PEPFAR-funded CAPRISA AIDS Treatment Programme (CAT) between June 2004 and August 2013. Patients were enrolled from two different catchment populations in KwaZulu-Natal; a TB clinic in the urban eThekwini district and a rural primary health care clinic in Vulindlela, rural KwaZulu-Natal. ART eligibility criteria, and definitions of immunologic and virological response to ART, defined as <400 copies/ml was as per current South African Government HIV/AIDS treatment guidelines [17]. Following ART initiation, patients presented for clinical review and adherence assessment weekly for the first two weeks, then monthly for the first 6 months, and every 3 months thereafter unless clinically indicated.

Routine demographic and clinical data were recorded at baseline and at follow-up visits. Laboratory safety assessments and CD4+ cell counts and viral loads were conducted at baseline and every 6 months or as clinically indicated. Patients were regarded as lost to follow-up if they missed 3 consecutive scheduled visits and if all attempts to track them telephonically and physically had failed. No lost to follow-up patients re-engaged in care. Patients provided written informed consent for participation in the CAT programme and for the use of their medical records. Approval for data collection and analysis was obtained from the University of KwaZulu-Natal, Biomedical Research Ethics Committee (Ref: E248/05).

### Data analysis

We used unpaired t-test, and Wilcoxon rank sum test for continuous data and Fisher’s exact test for categorical data to compare demographics and clinical characteristics between men and women. Data was censored at 72 months of follow-up. Kaplan-Meier was used to construct survival curves and compare them using the log-rank test. Poisson approximations and F-tests were used to construct 95% confidence intervals (CI) for mortality rate, TB incidence rate and rate ratios, respectively. We calculated age-standardised mortality rates using 5-year age band. The province of KwaZulu-Natal was used as a standard population. Predictors of mortality were assessed through both univariate and multivariate proportional hazards regression. Proportionality was assessed by fitting time dependent covariates in a model created by interacting baseline variables with survival time.

The association between gender and CD4+ cell count was determined through linear mixed models. All models were adjusted for baseline covariates such as clinic site, age, tuberculosis status at baseline, past history of TB, WHO stage, CD4+ cell count and viral load. We conducted sensitivity analysis evaluating the impact of ART programme maturity on the effect of gender on mortality during ART initiation periods of 2004–2008 and 2009–2013.

We calculated attributable risk in order to assess the relative contribution and impact of specific covariates on mortality. The effect of loss to follow-up on mortality was determined through sensitivity analysis. P values less than 0.05 were considered statistically significant. All analyses were conducted using SAS, version 9.4 (SAS Institute INC., Cary) and R version 3.2.2.

#### Role of the funding source

The funders of the study had no role in study design, data collection, data analysis, data interpretation, or writing of the report. The corresponding author had full access to all the data in the study and had final responsibility for the decision to submit for publication.

## Results

### Patient baseline clinical characteristics

A total of 4043 ambulant HIV infected patients, 1457 (36%) men and 2586 women (64%), 14 years and older, were initiated on ART between June 2004 and August 2013. Baseline clinical characteristics are presented in Table 1. Women comprised 59.4% and 40.6%, (p <0.001) of the study cohort at the urban and rural sites, respectively. Men were older, (median age of 35 vs 32 years), p<0.001, presented more often with WHO stage 4 disease (14.3% vs 9.7%), p<0.001 and were more likely to have had a past history of tuberculosis (TB) (34.4% vs 26.3%), p <0.001, (Table 1). At ART initiation, men had lower median CD4+ cell counts (113 vs131 cells/mm^3^), p <0.001, a lower median body mass index (BMI) (21.0 vs 24.2 kg/m^2^), p<0.001 and higher mean log viral loads (5.0 vs 4.9 copies/ml), p<0.001 (Table 1). The overall retention rate at 72 months was 91.8%, with 89.9% of men compared to 91.8% of women (p = 0∙07) retained in care. Men and women were loss to follow-up at a median (IQR) of 9.7 (2.7–23.7) and 12.6 (4.4–27.2) months respectively (p = 0.118). Rates of viral suppression, (89.5% vs 96%, p = 0.094) and proportion diagnosed with TB after ART initiation, (3.8% vs 4.2%, p = 0.620), did not differ significantly between men and women. In contrast, ART regimen change; 7∙1% vs 9.5% p = 0.012 was significantly different between men and women (Table 1).

**Table 1:** Baseline and follow-up characteristics of men and women initiated on ART.

Variable	Men (N = 1457)	Women (N = 2586)	p-value
***Characteristics at ART initiation***			
Overall age (years), median(IQR)	35 (30–41)	32 (28–38)	<0.001
**Age group**: **n (%)**			
<24	45 (3.1)	232 (9.0)	<0.001
24–34	655 (45.0)	1350 (52.3)	
35+	757 (52.0)	1001 (38.8)	
BMI(kg/m^2^), median(IQR)	21 (19.0–3.2)	24.2 (21.1–28.1)	<0.001
CD4+ cell count (cells/mm^3^), median(IQR)^a^	113 (47–177)	131 (68–189)	<0.001
**CD4+ cell count, cells/mm**^**3**^: **n (%)**			
<50	344 (25.9)	423 (18.3)	<0.001
50–200	783 (58.9)	1428 (61.6)	
>200	203 (15∙3)	466 (20.1)	
Log VL, mean(SD)^b^	5∙0 (0.9)	4.9 (0.9)	<0.001
Rural site, n (%)	692 (47.5)	1536 (59.4)	<0.001
Urban site, n (%)	765 (52.5)	1050 (40.6)	
WHO stage 1–3, n (%)^c^	1244 (85.7)	2315 (90.3)	<0.001
WHO stage 4, n (%)^c^	208 (14.3)	250 (9.7)	
Previous history of TB, n (%)^d^	483 (34.4)	661 (26.3)	<0.001
Prevalence of TB, n (%)	467 (32.1)	505 (19.5)	<0.001
***Follow-up***			
Number initiated on second line ART, n (%)	104 (7.1)	245 (9.5)	0.012
Lost to follow-up	161 (11.1)	239 (9.2)	0.070

^a^396 patents had missing baseline CD4+ count,

^b^496 missing VL,

^c^26 missing WHO stage,

^d^121 missing previous history TB

### Mortality

**Fig 1:**
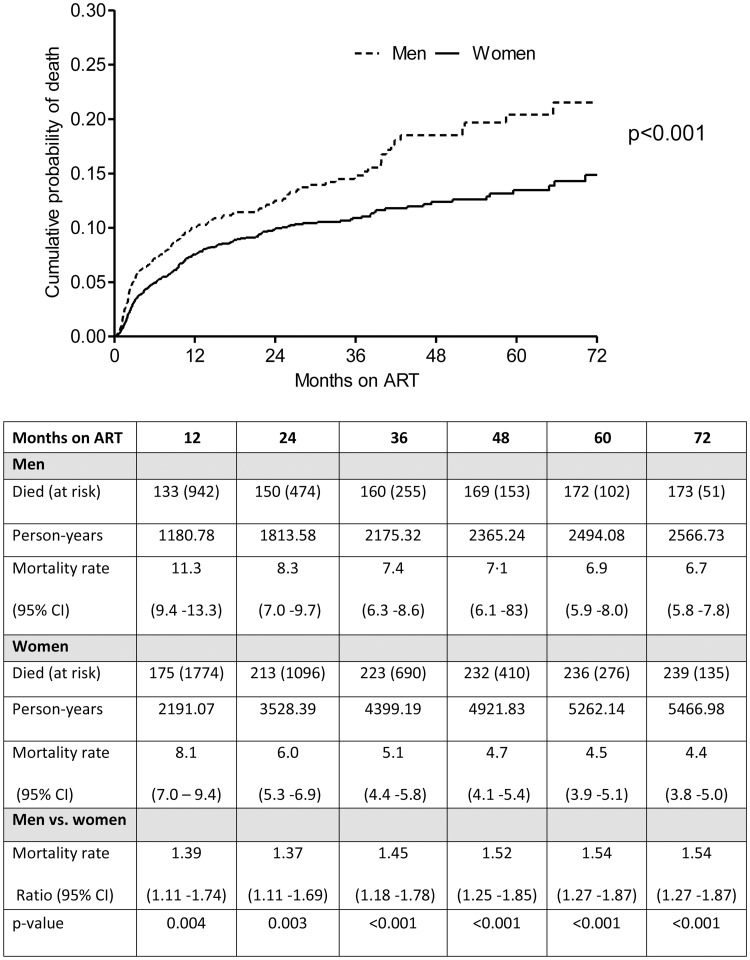
Kaplan-Meier estimates of cumulative annualised mortality rates between men and women accessing ART.

Deaths occurred among 173 men and 239 women, over 8033.71 person-years of follow-up, (median of 16.3 months [interquartile range (IQR), 8.1 to 35]), (Fig 1). Overall mortality rates were 5.1 per 100 person-years (p-y) (95% CI: 4.6–5.6) and 6.7 per 100 p-y (95% CI: 5.8–7.8) in men and 4.4 per 100 p-y (95% CI: 3.8–5.0) among women (mortality rate ratio: 1.54 (95% CI: 1.29–1.91, p< 0.001), (Fig 1). Age-standardised mortality rate was 7.9 per 100 p-y (95% CI: 4.1–11.7) for men and 5.7 per 100 p-y (95% CI: 2.7 to 8.6) for women (standardised mortality ratio: 1.38 (1.15 to 1.70). Mortality rates observed for men and women were significantly different at all follow-up time points up to 72 months; men had higher mortality rates throughout follow-up; peaking in the first 6 months post ART initiation at 15.2 (95% CI: 12.3–18.4) and 10.2 (95% CI: 8.5–12.2), between men and women respectively, p = 0.004 (Fig 1).

Mortality rates were highest in young men between the ages of 20 and 24 years at 11.8 per 100 p-y, (95% CI: 5.7–21.8) vs 3.9 per 100 p-y (95% CI: 2.4–5.9) in women of the same age, mortality rate ratio: 3.06 (95% CI: 1.45–6.46), p = 0.003 (Table 2). Among those ≥ 25 years, mortality rates among men were significantly different compared to women: 6.6 per 100 p-y (95% CI 5.6–7.7) vs 4.4 per 100 p-y (95% CI 3.9–5.1), mortality rate ratio: 1.49 (95% CI: 1.21–1.83), p <0.001 (Table 2). The mortality rate between men and women stratified by baseline CD4+ cell count was 10.7 (95% CI 8.3–13.6) and 8.2 (95% CI 6.5–10.1) for patients with baseline CD4+ cell counts <50 cells/mm^3^, p = 0.098; 5.4 (95% CI 4.3–6.7) and 3.8 (95% CI 3.2–4.6) for baseline CD4+ cell counts of 50–200 cells/mm^3^, p = 0.012 and 5.2 (95% CI 2.8–8.9) and 2.1 (95% CI 1.2–3.3). Mortality rate ratio stratified by baseline CD4+ cell count comparing men and women was 1.31 (95% CI 0.95–1.8) for baseline CD4+ cell counts < 50 cells/mm^3^, p = 0.098; 1.42 (95% CI 1.08–1.87) for baseline CD4+ cell counts of 50–200 cells/mm^3^, p = 0.012 and 2.1 (95% CI 1.22–5.26) for baseline CD4+ cell counts of > 200 cells/mm^3^ (Table 2).

**Table 2:** Mortality rates for men and women stratified by baseline CD4+ cell count and age.

	Men			Women				
Characteristic	No of deaths	Person-years	Mortality rate (95% CI)	No of deaths	Person-years	Mortality rate (95% CI)	Rate ratio (95% CI)	p-value
**Age group (years)**								
**<20**	1	16.32	6.1 (0.2–34.1)	3	86.31	3.5 (0.7–10.2)	1.76 (0.18–16.92)	0.624
**20–24**	10	84.42	11.8 (5.7–21.8)	22	568.07	3.9 (2.4–5.9)	3.06 (1.45–6.46)	0.003
**≥25**	162	2460.88	6.6 (5.6–7.7)	213	4808.60	4.4 (3.9–5.1)	1.49 (1.21–1.83)	<0.001
**Baseline CD4+ count (cells/mm**^**3**^**)**								
**<50**	67	626.59	10.7 (8.3–13.6)	85	1039.12	8.2 (6.5–10.1)	1.31 (0.95–1.8)	0.098
**50–200**	85	1563.29	5.4 (4.3–6.7)	128	3343.48	3.8 (3.2–4.6)	1.42 (1.08–1.87)	0.012
**>200**	13	250.01	5.2 (2.8–8.9)	16	777.17	2.1 (1.2–3.3)	2.53 (1.22–5.26)	0.013

### Treatment response

**Fig 2:**
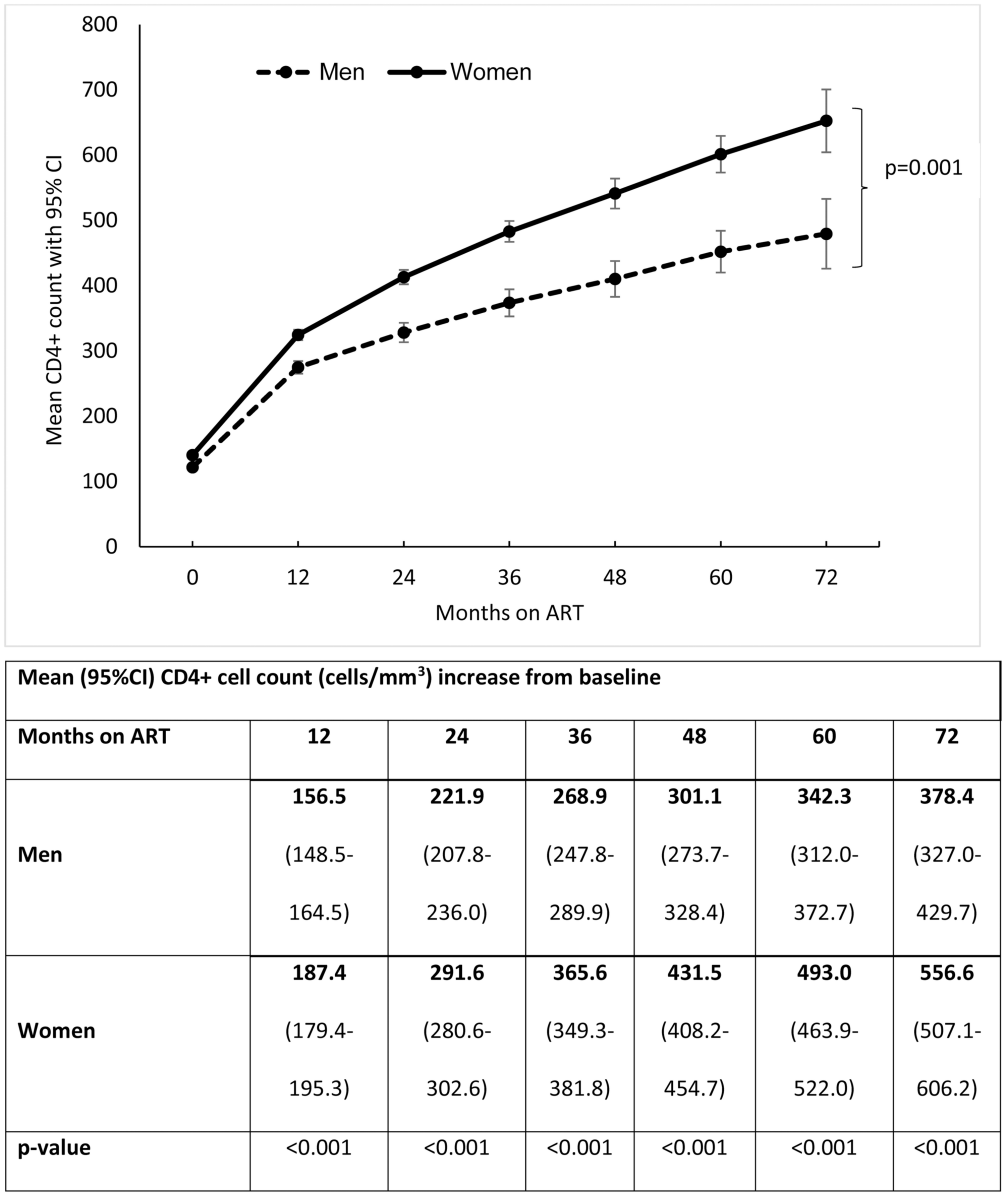
Mean CD4+ cell count change from baseline over time.

Women demonstrated a better immunological response during ART follow-up showing consistently higher mean CD4+ cell counts at each clinical assessment (Fig 2). Mean CD4+ cell count increases post-ART initiation were lower in men at all follow-up time points (Fig 2). At 72 months of ART, mean CD4+ cell count gain was 378.4 cells/mm^3^ and 556.6 cells/mm^3^, p = 0.001 among men and women respectively (Fig 2). Multivariate linear mixed model showed a significant interaction between gender and follow-up time suggesting significantly different CD4+ cell count recovery between men and women; p = 0.001 (Fig 2).

Adjusted proportional hazards regression analysis showed that men had a 29% higher risk of death compared to women (adjusted hazard ratio (aHR): 1.29, 95% CI: 1.05–1.58, p = 0.016 (Table 3). Risk of mortality varied by pre-ART CD4+ cell count. Compared to patients with baseline CD4+ cell counts >200 cells/mm^3^, there was a 4-fold higher risk of death among patients with a baseline CD4+ cell count <50 cells/mm^3^ (aHR: 3.86, 95% CI: 2.55–5.84), p<0.0001, and a two-fold higher risk of death among patients with a baseline CD4+ cell count between 50–200 cells/mm^3^, (aHR: 1.94, 95%CI: 1.30–2.91), p = 0.001, (Table 3).

**Table 3:** Analysis of factors associated with mortality.

Variable at ART initiation		Univariate		Multivariate	
		HR (95% CI)	p-value	HR (95% CI)	p-value
Gender (ref: women)	Male	1.41 (1.16–1.72)	0.001	1.29 (1.05–1.58)	0.016
Age (per 5 year increase)		1.03 (0.97–1.08)	0.364	1.04 (0.98–1.10)	0.182
Site (ref: rural)	Urban	0.82 (0.67–1.01)	0.061	0.90 (0.70–1.16)	0.410
Baseline CD4+ cell count (ref:>200), cells/mm^3^	<50	4.10 (2.75–6.10)	<.0001	3.86 (2.55–5.84)	<.0001
	50–200	1.89 (1.28–2.79)	0.001	1.94 (1.30–2.91)	0.001
Viral load (log_10_ copies/ml)		1.52 (1.31–1.76)	<0.001	1.30 (1.11–1.52)	0.001
Tuberculosis (ref: Yes)	No	1.26 (0.98–1.61)	0.0711	1.74 (1.33–2.28)	<.0001
WHO stage (ref: 1–3)	Stage 4	2.49 (1.97–3.15)	<.0001	2.32 (1.80–2.99)	<.0001
Past history of TB (ref: No)	Yes	1.20 (0.97–1.47)	0.092	1.04 (0.81–1.33)	0.759

In addition, patients presenting with no TB at ART initiation or a higher mean log viral load or WHO stage 4 disease had significantly higher risk of death (Table 3).

We found that the attributable risk of death among patients initiating ART was 24.3% (95% CI 17.3–30.5) for patients with a low baseline CD4+ cell count and 10.2% (95% CI 2.7–16.9) for male patients (Table 4).

**Table 4:** Impact of different risk factors on mortality.

Variable	Attributable risk (95% CI)
Male	10.2% (2.7–16.9)
CD4+ cell count <50cells/mm^3^	24.3% (17.3–30.5)
WHO stage 4	13.2% (9.0–18.1)
No TB prevalence	24.2% (7.0–40.8)

Of note, sensitivity analysis evaluating the impact of ART programme maturity on the effect of gender on mortality showed that mortality rates between men and women was consistently two-fold higher and significantly different at all follow-up time-points between 2009–2013. This finding did not hold for the period 2004–2008 during the period of ART initiation (Table 5).

**Table 5:** Sensitivity analysis: Mortality rate by gender.

	Men			Women				
Follow-up	No of deaths	Person-years	Mortality rate (95% CI)	No of deaths	Person-years	Mortality rate (95% CI)	Mortality Rate ratio (95% CI)	p-value
Scenario†	334	2566.73	13.0 (11.7–14.5)	478	5466.98	8.7 (8.0–9.6)	1.49 (1.30–1.71)	<0.001
Scenario‡	236	2566.73	9.2 (8.1–10.4)	316	5466.98	5.8 (5.2–6.5)	1.59 (1.34–1.88)	<0.001

^†^ Assumes all patients who were loss to follow-up died

^‡^ Assumes all patients who were loss to follow-up within 6 months of ART initiation died

## Discussion

We provide evidence of gender disparities between HIV infected men and women in access and response to antiretroviral therapy. Clinical outcomes differed by gender; with men demonstrating higher overall mortality rates and suboptimal immunologic recovery.

### Mortality

The bulk of deaths in both men and women occurred in the first 24 months of follow-up concurring with findings from other studies [18,19]. Despite ART access, all patients showed unacceptably high mortality rates with men having 50% higher mortality rate compared women throughout follow-up and their mortality was consistently higher irrespective of baseline CD4+ cell counts. Results from sensitivity analyses, which assumed that all patients who were lost to follow-up died, and that all patients who were lost to follow-up within six months of ART initiation died, were consistent with the main findings (S1 Table).

After removing the confounding effect of age, mortality rates observed in men and women in our study was lower than the age-standardised mortality rates. Interestingly, mortality rates among men and women in the study population closely approximated age-standardised mortality when we assumed that patients who were loss to follow-up within 6 months of ART initiation died.

In contrast to the epidemiological data of the evolving epidemic in southern Africa wherein HIV acquisition does not occur in men until their late twenties [20,21] and we found that the highest mortality rates were in young men between the ages of 20–24 years. However, we had a very small number of men less than 24 years of age enrolled into the programme.

Conversely, studies from developed countries show differing results, finding no gender differences in clinical, virological and immunological outcomes among HIV infected men and women initiating ART [22,23]. A multi-site cohort study found higher rates of virological rebound in women [7]. Studies conducted elsewhere in SSA also demonstrate poor outcomes among men on ART with respect to higher rates of mortality and loss-to-follow-up [24,25]. Published literature from South Africa support our findings and demonstrates that while life expectancy was not significantly different pre-ART between genders; in 2011 women were 27% less likely to die from HIV than men [26].

### Risk factors for poor clinical outcomes

Poor prognostic markers of ART response were apparent in men at baseline, who were older, had lower CD4+ cell counts and higher viral loads compared to women. This finding concurs with published South African literature demonstrating that compared to men, women were more likely to access HIV testing, and among those testing HIV positive, women were younger with higher CD4+ cell counts than their male counterparts [27].

This study also showed that the late presentation of men was associated with poor immunological response to ART with higher risk of morbidity and mortality [27]. Our findings of significant correlation between baseline characteristics, virological suppression rates, immunologic recovery and mortality concur with previously published studies conducted in South Africa [28,29]. There were no significant differences in virologic suppression in follow-up between men and women. Notwithstanding the greater than 90% virologic suppression rate on ART, men remained at higher risk of dying, emphasising the impact of advanced HIV disease at baseline on subsequent antiretroviral treatment response. We found significantly different CD4+ cell count recovery between men and women with men consistently demonstrated significantly smaller CD4+ cell count increases compared to women during longitudinal follow-up. Given that viral load suppression was similar between sexes, women however had higher CD4+ cell counts indicating that there may be underlying genetic differences in ART response.

We show that during follow up overall mortality rate halved among men and women with baseline CD4+ cell counts of 50–200 cells/mm^3^ in comparison with those patients that presented with baseline CD4+ cell counts of less than 50 cells/mm^3^. Furthermore, we found that the mortality risk among patients initiating ART was attributable to both low baseline CD4+ cell count and male gender, corroborating published literature showing that baseline CD4+ cell count is an important factor impacting CD4+ cell count increase and recovery on treatment [30]. In settings that are still guided by CD4+ cell count thresholds for ART initiation consideration should be given for men to access ART at higher CD4+ cell counts.

Our findings demonstrate that the absence of a diagnosis of prevalent TB increased risk of mortality. Published literature has cited that undiagnosed TB is higher among patients accessing ART than in the general population; with the majority of incident TB diagnosed in the early weeks of ART initiation being TB prevalent but missed at baseline screening [19,31,32]. It is possible that undiagnosed TB at ART initiation also contributed to higher risk of mortality in our study. The magnitude of the TB epidemic, burden of disease and varied presentation of TB in HIV infected individuals may have contributed to missed TB diagnosis.

### Programmatic outcomes

Men had higher lost to follow-up rates in our cohort in keeping with reports from published literature [33,34]. Adverse clinical outcomes notably death can be masked by LTFU resulting in the underestimation of actual mortality rates within ART programmes [35]. Previous South African studies have documented gender differences in service uptake within the HIV care cascade; women have higher rates of HIV testing [27] and linkage to care than men [13]. Once initiated on ART, men have poorer adherence [36] and lower retention in care [28,35]. Govindasamy et al in a systematic review of 42 publications from 12 countries showed high attrition of men from pre-ART and ART programmes [37]. These studies did not however describe mortality differentials between the genders. Overall, the design and results of this article are similar to those found by Cornell et al [28].

### Limitations

As this was a secondary analysis of routinely collected programme data, missing data limited our ability to comment on various correlations e.g. the impact of socio-economic and demographic variables on clinical outcomes. Treatment adherence was not measured and its impact on outcomes could be underestimated. We have not investigated other biological and behavioural factors that may have contributed to poor ART outcomes in men. While the vast majority of patients enrolled into this cohort were women, we had very few pregnancies and are therefore unable to comment on the impact of pregnancy in women on mortality in this cohort. Additional studies that explore potential barriers that South African men experience when seeking HIV care, and optimal strategies to link and engage men in ART services, is warranted.

## Conclusion

While heterosexual transmission is the primary mode of HIV transmission in sub Saharan Africa, the impact of free ART provision on access and clinical outcomes in men and women is unequal. Men presented late for ART initiation with more advanced disease and a higher mortality rate compared to women in this six-year ART program. The increased feminization of the HIV epidemic in sub Saharan Africa has rightfully demanded that greater focus be given to women with respect to HIV prevention, transmission, treatment and care. However, in a predominantly heterosexual epidemic, greater programmatic effort should be directed toward encouraging men to test early and frequently. As we prepare to scale-up “Test and Treat” in all HIV infected patients, innovative strategies are needed to ensure that men and women that test HIV positive have access to effective linkage to care for early ART initiation to achieve the goal of reducing mortality and increasing AIDS free survival.

## Supporting information

S1 Table (DOCX)

S2 Table (DOCX)
