# Supplementary material for: High mortality rates in men initiated on anti-retroviral treatment in KwaZulu-Natal, South Africa
Source: PLoS One. 2017 Sep 13;12(9):e0184124. doi: 10.1371/journal.pone.0184124 (PMC5597205; doi:10.1371/journal.pone.0184124)
Supplement: S1 Table — (DOCX) [file pone.0184124.s001.docx]

**S1 Table: Sensitivity Analysis: Mortality rate for men and women stratified by ART period**

|  | **2004-2008** | | | | | | | | **2009-2013** | | | | | | | |
| --- | --- | --- | --- | --- | --- | --- | --- | --- | --- | --- | --- | --- | --- | --- | --- | --- |
|  | **Men** | | | **Women** | | |  | | **Men** | | | **Women** | | |  |  |
| **Follow-up** | **Deaths** | **Person years** | **Mortality rate**  **(95% CI)** | **Deaths** | **Person years** | **Mortality rate**  **(95% CI)** | **Mortality rate ratio (95% CI)** | **p-value** | **Deaths** | **Pearson years** | **Mortality rate**  **(95% CI)** | **Deaths** | **Person years** | **Mortality rate**  **(95% CI)** | **Mortality rate ratio (95% CI)** | **p-value** |
| Month6 | 46 | 252∙40 | 18∙2  (13∙3 -24∙3) | 79 | 574∙72 | 13∙7  (10∙9 -17∙1) | 1∙33  (0∙92 -1∙91) | 0∙124 | 54 | 406∙90 | 13∙3  (10∙0 -17∙3) | 45 | 637∙64 | 7∙1  (5∙1 -9∙4) | 1∙88  (1∙27 -2∙79) | 0∙002 |
| Month12 | 59 | 480∙64 | 12∙3  (9∙3 -15∙8) | 118 | 1098∙49 | 10∙7  (8∙9 -12∙9) | 1∙14  (0∙83 -1∙56) | 0∙411 | 74 | 700∙14 | 10∙6  (8∙3 -13∙3) | 60 | 1092∙58 | 5∙5  (4∙2 -7∙1) | 1∙92  (1∙37 -2∙7) | <0∙001 |
| Month24 | 69 | 879∙57 | 7∙8  (6∙1 -9∙9) | 144 | 2049∙02 | 7∙0  (5∙9 -8∙3) | 1∙12  (0∙84 -1∙49) | 0∙439 | 81 | 934∙01 | 8∙7  (6∙9 -10∙8) | 69 | 1479∙36 | 4∙7  (3∙6 -5∙9) | 1∙86  (1∙35 -2∙56) | <0∙001 |
| Month36 | 76 | 1171∙20 | 6∙5  (5∙1 -8∙1) | 153 | 2793∙41 | 5∙5  (4∙6 -6∙4) | 1∙18  (0∙9 -1∙55) | 0∙238 | 84 | 1004∙11 | 8∙4  (6∙7 -10∙4) | 70 | 1605∙78 | 4∙4  (3∙4 -5∙5) | 1∙92  (1∙4 -2∙64) | <0∙001 |
| Month48 | 84 | 1353.59 | 6.2  (4.9 -7.7) | 162 | 3297.58 | 4.9  (4.2 -5.7) | 1∙26  (0.97 -1.64) | 0∙086 | 85 | 1011∙64 | 8.4  (6∙7 -10∙4) | 70 | 1624∙25 | 4.3  (3∙4 -5∙4) | 1.95  (1∙42 -2∙68) | <0∙001 |
| Month60 | 87 | 1481.71 | 5.9  (4.7 -7.2) | 166 | 3637.50 | 4.6  (3.9 -5.3) | 1∙29  (1∙0 -1.67) | 0∙054 | 85 | 1012∙37 | 8.4  (6∙7 -10∙4) | 70 | 1624∙64 | 4.3  (3∙4 -5∙4) | 1.95  (1∙42 -2∙68) | <0∙001 |
| Month72 | 88 | 1554.36 | 5.7  (4.5 -7.0) | 169 | 3842.35 | 4∙4  (3∙8 -5∙1) | 1∙29  (1∙0 -1∙67) | 0∙053 | 85 | 1012∙37 | 8.4  (6∙7 -10∙4) | 70 | 1624∙64 | 4.3  (3∙4 -5∙4) | 1.95  (1∙42 -2∙68) | <0∙001 |
