# Supplementary material for: High mortality rates in men initiated on anti-retroviral treatment in KwaZulu-Natal, South Africa
Source: PLoS One. 2017 Sep 13;12(9):e0184124. doi: 10.1371/journal.pone.0184124 (PMC5597205; doi:10.1371/journal.pone.0184124)
Supplement: S2 Table — (DOCX) [file pone.0184124.s002.docx]

**S2 Table 2: TB incidence rate for men and women**

|  | **Men** | | | **Women** | | |  | |
| --- | --- | --- | --- | --- | --- | --- | --- | --- |
| **Follow-up** | **TB cases** | **Person-years** | **TB incidence rate**  **(95% CI)** | **TB cases** | **Person-years** | **TB incidence rate**  **(95% CI)** | **Rate ratio**  **(95% CI)** | **p-value** |
| Month 6 | 28 | 612.56 | 4.6 (3.0 -6.6) | 53 | 1160.14 | 4.6 (3.4 -6.0) | 1.00 (0.63 -1.58) | 1.000 |
| Month 12 | 38 | 1063.55 | 3.6 (2.5 -4.9) | 75 | 2042.23 | 3.7 (2.9 -4.6) | 0.97 (0.66 -1.43) | 0.878 |
| Month 24 | 48 | 1606.02 | 3.0 (2.2 -4.0) | 95 | 3241.45 | 2.9 (2.4 -3.6) | 1.02 (0.72 -1.44) | 0.911 |
| Month 36 | 54 | 1921.28 | 2.8 (2.1 -3.7) | 103 | 4024.29 | 2.6 (2.1 -3.1) | 1.10 (0.79 -1.53) | 0.571 |
| Month 48 | 55 | 2087.20 | 2.6 (2.0 -3.4) | 106 | 4495.54 | 2.4 (1.9 -2.9) | 1.12 (0.81 -1.55) | 0.495 |
| Month 60 | 56 | 2197.66 | 2.5 (1.9 -3.3) | 109 | 4801.62 | 2.3 (1.9 -2.7) | 1.12 (0.81 -1.55) | 0.491 |
| Month 72 | 56 | 2258.31 | 2.5 (1.9 -3.2) | 109 | 4984.16 | 2.2 (1.8 -2.6) | 1.13 (0.82 -1.56) | 0.457 |
| Overall | 56 | 2258.31 | 2.5 (1.9 -3.2) | 109 | 4984.16 | 2.2 (1.8 -2.6) | 1.13 (0.82 -1.56) | 0.457 |
